# Professional Brotherhood

**Published:** 1871-04

**Authors:** 


### PART IV.

### Editorials, Reviews, Items and News.

### PROFESSIONAL BROTHERHOOD.

The time draws near, and is even already at hand, when we trust large numbers of our professional brethren, who have been so fortunate in the last twelve months as to earn a fair living, to provide food and shelter and a few nice things over for the wife and children, are clear-headed and light-hearted enough to be packing satchel for the reunion with the brethren of the State and National Medical Associations; we can see them converging by the main lines of travel to the great starting point on the Union and Pacific Road. As the corteries formed on different routes are brought together at leading junctions, we can see the spirit of joyous merriment, of hearty clasping of hands by old friends and veterans who have come together to recount another year's triumphs and vicisitudes. We see all these corteries gathered at Omaha on a given day, at a given hour, to commence the grand journey. We may justly call it across the valley, across the plains—through the mountains and down the Pacific slope, to the great, the only city of our Pacific coast. They are all free and happy.

The cares of private business and professional engagements have been provided for as well as human foresight could devise; all that is left behind is dismissed with a trust in Providence, and for once again in each year each traveller gives up his heart and mind to free and unrestrained intercourse and thought with his fellow and professional brother. Who would not delight to be there with them? We hope thousands will avail themselves of this indescribable pleasure, and we hope to receive from some graphic pen interesting reports of much of the wit and humor as well as the most striking incidents of the travel.

The influence of such scenes and such reunions as we have ventured to anticipate are certainly favorable to the cultivation and outgushing of fraternal feelings. Certainly fraternal feeling in the medical profession should have a rallying point—a grand centre somewhere. We have been among our professional brethren on more than one of these occasions, and we do know that then the current of true fraternal feeling—of real professional fellowship flowd free, and clear, and strong. It is worth seeing once, if never again, just to enjoy it. We commend the experiment to all who doubt. That will settle the question, and prove that there is, now and then, manifested—when developed by favorable causes—and that there always exists, even when crowded down by the many hateful obstacles to its generation and culture—a spirit of brotherhood in the medical profession. This leads us to inquire why this spirit manifests itself so little at home, so little locally, and so readily abroad? There are many causes, indeed too many, here to recount. On these joyous anniversaries our brethren are happy and fraternal, not only because free for the moment from private and professional cares and engagements, but more because they are free from those local jealousies and hatreds and rivalries in the profession.

Whence arise these local and personal professional hostilities and antagonisms?—these real eating cancers in the body of professional brotherhood? Whence do they arise? They are everywhere, and they are legion. We submit these inquiries to our brethren soon to assemble in National or State Associations. Their position, their advantages as delegates are all highly favorable to the tracing out and thoroughly understanding the cause of the evil and prescribing the remedy which we believe can only be found in organization—everywhere a hearty co-operating spirit, a whole-soul brotherhood, true fraternal union, blending professional hearts into one grand pulsation, for the good of all; organization, true to its principles, cementing the disciples of peace, and frowning upon all schisms and schismatics.

What an army with banners—what a terror to all its foes, armed with such an organization; what a power for good, if its members only knew the value of that maxim, "In union there is strength"—a maxim trite indeed, yet among all the fruits of human wisdom as eminent and as practical as the richest of good results. Let us contemplate it in connection with our profession. In Society, in State, or Church, dissensions beget weakness, and a divided force achieves no success. It is sad to contemplate how lamentably this has been exemplified in the world's history—how much of enthusiasm and vital energy have been wasted for lack of united effort. The most melancholy records of human life are those in which are recounted the clashings of elements that should have been united, and the contentions of those who ought to have felt and acted as brethren. It is painful to think how greatly the cause of such has suffered and how much human progress has been checked by this want of united exertion.

All will admit that a want of harmony in church or state is deplorable ; but it is not equally apparent that discord among men of science results disastrously. It would seem, indeed, that a conflict of theories would be more likely to elicit truth, and therefore rather to be desired than deprecated. Such is the case where the matter is purely speculative. There may be subjects upon which theorists have expended the force of their intellects in which the largest amount of union would do no good, nor the fiercest discord any harm. But if the question of science be one of practice, upon which much of the happiness of our race depends, then the more closly its professors are agreed, the greater good may they accomplish. The differences among physicians have passed into a proverb, and have for generations been a subject of ridicule. They are, however, matters for grief rather than for mirth, for it would surpass the powers of human calculation to estimate the suffering of which these differences have been the cause. It has been through these that empiricism has been enabled to inflict its blighting influences and woes upon mankind. We may guess, but we can never know, how much more the cause of true medical science would have been advanced had all who labored for it labored together. We would not have professional men harmonize at the expense or sacrifice of principle. Truth should never, in the keeping of her votaries, be humiliated and disgraced by compromising its purity with the garbish of error; but its claims should be asserted and vindicated against the combined powers of darkness—even at the sacrifice of the tenderest emotions of the heart or the severing of the sweetest endearments of personal friendship. There is an element in every pure heart, an implantation of deity, which fits it for the highest and noblest duties and obligations of life—a spirituality which stamps the soul as plastic material out of which shall be wrought the richest treasures of fraternal kindness and love. Strip the human heart of this—drain it of its spiritual gold, and the dross of its corruption will linger to pollute the memories of the past and the hopes of the future.

No man can be considered good material for the foundation of a real trustworthy brotherhood who would, for any consideration, from personal friendship or blood connection exert himself to make, or aid others in making, wrong appear right at the sacrifice of justice and character. None but those who know the law and who obey it can be trusted. Every scientific man should vie with every other one of his profession in the struggle for excellcene. He should feel it his duty to employ the best powers of his intellect in tracing out and applying the principles of science for the relief of the suffering; but while he is ever cautiously regardful of the duties which he owes to his patients, he must be equally mindful of the duties which he owes to himself and his brethren. To himself he should feel bound at all times to exhibit the character of an honest, high-toned, truthful gentleman, scorning all attempts to deceive either for the sake of emolument or for the sake of honor; to his brethren it is due that he bear himself with the politeness of a generous rival, regarding their reputation as dearly as his own, and studiously refraining from everything that would bear the slightest appearance of an effort to supplant. His feeling of brotherhood should prompt him to display towards them, a kindness and consideration surpassing the politeness of society. It should produce something more than gentlemanly courtesy—more than the good feeling induced by fellowship. He should feel for the interest and welfare of those of his profession almost as he would feel for his own. While in the struggle for the accomplishment of the greatest good he is each one's rival, it is a high-toned emulation which will neither underrate others nor overate himself. For such a brotherhood to exist, there must be terms of admission and rules for government. No profession, however catholic in its tendencies, could confer the rights and privileges of its communion upon all who might choose to claim them, irrespective of qualification. This principle finds a practical illustration in the safeguards thrown around the various Christian denominations, for the purpose, as supposed by them, of protecting their organizations from conflict of opinions and subversion of their tenets. Every denomination asserts its right to determine the character and qualifications of those applying for membership, and in enforcing the terms by which such connection shall be perpetuated. Our junior editor is a Baptist. He would be untrue to the principles and tenets of his articles of faith should he advocate and practice "open communion." And while every Baptist of like "faith and order" can be welcomed to the "Table of the Lord," who is in good standing, he cannot himself commune with those, or allow others to commune with him, who do not obey that rule or are outside of his church. The senior is a Methodist; he believes all Christians should be allowed to sit at the same "Table of the Lord." He does not go beyond his Christian brethren to invite outsiders to partake of the sacrament; the Christian alone is invited. While he does not make war upon his junior because of his adherance to the law of his denomination, he cannot claim religious recognition and affiliation with him, no more than the senior would those who are not connected with any denomination, and hence refused recognition and affiliation, in this respect, with himself.

So a physician, before he accords the right of professional brotherhood, should be satisfied that he can rightfully claim them. This claim must not rest solely on the fact of his having studied medicine and graduated at a school of high standing. Many men have done this, and have made no mean attainments in science, who do not deserve to be ranked as members of the brotherhood. In addition to having acquired a certain amount of knowledge, he must become in other respects what the profession requires of its members. He must in every essential point conform to those rules which have been adopted for their government. If he set these at naught he must be reckoned an outsider, whatever may be his age, influence, natural gifts, or his acquirements. The brotherhood should no more respect his claims to be one of their number, or entitled to professional recognition by any member who is in full fellowship, than a church would retain one who refused assent to her cardinal doctrines, and lived in open disregard of her most positive requirements, or those who sustained him and endorsed his rebellious course. Men may differ on many points, and yet be harmonious. Theologians may differ in the interpretation of texts, and yet love each other as brethren. But if the point of difference be a cardinal item of their respective creeds, they can avoid dissension only by non-communion. So Physicians may disagree as to the treatment of fever or the use of the lancet, and still exhibit no lack of confidence or want of courtesy. If, however, it is a question upon which the whole theory of the profession is based, there can be no affiliation between them. The scientific physician, who is in earnest for the cause of truth, and zealous for the dignity of his calling, can hold no fellowship with impirics who prostitute the knowledge which they may possess to the base purpose of imposing on popular credulity, and who have no higher aim than amassing fortune out of human suffering.

It is really a question of humanity that the medical profession should have laws by which its members shall be governed, and that these laws be rigidly enforced. It is essentially a benevolent profession. It addresses itself to the suffering. It is, therefore, a matter of the very first moment that the afflicted should know to whom they should apply. The medical fraternity have assumed the duty of indicating the sources whence relief may be sought, and the assumption is one of fearful responsibility. There be many who make great pretentions—who offer to the children of affliction life and health at a slight sacrifice of convenience and money. Are they worthy of confidence? Do they possess in reality such power over disease as they claim? Is their practice the legitimate application of scientific truths, or is it mere guess work? or, worse still, mere quackery? These are grave questions, propounded by the public to the medical fraternity, and on their reply hang momentous issues. If all who profess to heal are beloved and recognized members of that fraternity, then is it responsible for their practice and the harm that may be done by them may be, in a degree, chargeable to all. Considered in this aspect, is it not of the very first importance that the Brotherhood of Physicians should have and maintain laws as to who shall codtitute its members, and how those members shall be governed and controlled?

